# SMARCAL1 Negatively Regulates C-*Myc* Transcription By Altering The Conformation Of The Promoter Region

**DOI:** 10.1038/srep17910

**Published:** 2015-12-09

**Authors:** Tapan Sharma, Ritu Bansal, Dominic Thangminlen Haokip, Isha Goel, Rohini Muthuswami

**Affiliations:** 1 School of Life Sciences, JNU, New Delhi 110067

## Abstract

SMARCAL1, a member of the SWI2/SNF2 protein family, stabilizes replication forks during DNA damage. In this manuscript, we provide the first evidence that SMARCAL1 is also a transcriptional co-regulator modulating the expression of c-Myc, a transcription factor that regulates 10–15% genes in the human genome. BRG1, SMARCAL1 and RNAPII were found localized onto the c-*myc* promoter. When HeLa cells were serum starved, the occupancy of SMARCAL1 on the c-*myc* promoter increased while that of BRG1 and RNAPII decreased correlating with repression of c-*myc* transcription. Using Active DNA-dependent ATPase A Domain (ADAAD), the bovine homolog of SMARCAL1, we show that the protein can hydrolyze ATP using a specific region upstream of the CT element of the c*-myc* promoter as a DNA effector. The energy, thereby, released is harnessed to alter the conformation of the promoter DNA. We propose that SMARCAL1 negatively regulates c-*myc* transcription by altering the conformation of its promoter region during differentiation.

ATP-dependent chromatin remodeling proteins regulate gene expression either by repositioning nucleosomes or by incorporating histone variants into the nucleosomes^1^,^2^,^3^. Baradaran-Heravi *et al*. have proposed a third possibility wherein they postulated that SMARCAL1, a distant member of the ATP-dependent chromatin remodeling protein family, could be regulating transcription of genes such as c-*myc* and c*-kit* by altering DNA structure in an ATP-dependent manner^4^. SMARCAL1 is a 105-kDa protein that hydrolyses ATP only in the presence of DNA molecules containing double-strand to single-strand transition regions^5^,^6^,^7^,^8^. *In vivo*, upon DNA damage the protein is recruited by RPA to stalled replication forks^9^,^10^. SMARCAL1 stabilizes the stalled replication forks due to its annealing helicase activity, thus playing an important role in maintaining genome stability^9^,^10^,^11^,^12^,^13^,^14^. Mutations in SMARCAL1 have been linked to the autosomal recessive disorder, Schimke Immunoosseous Dysplasia (SIOD)^15^. Patients afflicted with SIOD exhibit a wide range of phenotypes including skeletal dysplasia, T-cell immunodeficiency and renal dysfunction leading Boerkoel *et al*. to hypothesize that SMARCAL1 could be a transcriptional regulator of a subset of genes, both during and after development^15^,^16^. Experiments using zebra fish model system have shown that knockdown of *SMARCAL1* causes multi-system developmental abnormalities affecting gene expression of *gata1*, *beta-E1 globin* and other genes^17^. Recent studies have also shown that gene expression profile is altered in *SMARCAL1*^−/−^ mice, supporting the hypothesis that the protein could function as a transcription regulator^4^.

The transcription factor c-*myc*, a leucine zipper protein, regulates the expression of 10–15% of human genes, thus playing an important role in cell proliferation, differentiation, growth and survival; overexpression of the protein is associated with cancer^18^,^19^,^20^,^21^. The c-*myc* gene is exquisitely controlled and its expression is fine-tuned by many transcription factors^22^. The gene contains multiple promoters; in human cells four promoters have been documented: P0, P1, P2, and P3 with P2 being the maximally used promoter^21^,^23^. A GC-rich region, known as CT element, present −142 to −115 bp upstream of the P1 promoter, is the major regulator of c-*myc* expression by the formation of G-quadruplex and I-motif^24^,^25^,^26^. In addition to the CT element, a Far UpStream Element (FUSE) present 1.7 kb upstream of the P1 promoter has also been identified^27^. BRG1, an ATP-dependent chromatin remodeling protein, has been shown to remodel the nucleosomes around the FUSE region when cells are released from serum starvation^28^,^29^.

In this paper, we have explored the role of BRG1 and SMARCAL1 in regulating the expression of c-*myc*. We have shown that both BRG1 and SMARCAL1 bind to a 159 bp DNA segment upstream of the CT element which will be referred to as Myc_B_159_ in the remaining manuscript. Activation of c-*myc* gene was dependent on binding of BRG1 and RNA polymerase II (RNAPII) to Myc_B_159_. In contrast, binding of SMARCAL1 to this region of the c-*myc* promoter led to repression of c-*myc* transcription. Using ADAAD, the bovine homolog of SMARCAL1, we have shown that ADAAD binds to Myc_B_159_ with an apparent K_M_ of 3.6 ± 0.3 nM. CD spectroscopy showed that ADAAD-Myc_B_159_ interaction results in alteration in the conformation of DNA in an ATP-dependent manner. We found that SMARCAL1 regulates differentiation of K562 cells in response to phorbol myristate acetate (PMA) by transcriptionally repressing c*-myc* expre transcriptionally repressing c-myc expression leading us to leading us to propose that the phenotypic manifestation of SIOD could be due to the changes in gene expression profiles of key transcription factors which are directly or indirectly regulated by SMARCAL1 with the negative regulation of c-*myc* presented in this paper being one such example.

## Results

### Downregulation of *SMARCAL1* leads to altered gene expression pattern

Baradaran-Heravi *et al*. have hypothesized that SMARCAL1 can possibly regulate genes like c-*kit* and c*-myc* by altering the promoter structure^4^. c-*kit* expression is regulated by G-quadruplex formation, a feature that is shared by another transcription factor, c-*myc*, which regulates 10–15% of genes in mammalian cells. To explore whether SMARCAL1 can regulate gene expression of c-*myc*, we downregulated *SMARCAL1* in HeLa cells using shRNA and obtained three monoclonals- Sh1, Sh2, and Sh3 as well as one polyclonal cell line (Sh). We confirmed that SMARCAL1 was indeed downregulated in all these cell lines using quantitative real-time RT-PCR (Supplementary Fig. S1). Since BRG1 is also known to regulate the transcription of c-*myc* by binding to the FUSE region^29^, and SMARCAL1 regulates *brg1* expression (Haokip *et al*. companion paper) we analyzed the expression of c*-myc* and *brg1*. We found that both *brg1* (Supplementary Fig. S1) as well as c-*myc* were downregulated (Supplementary Fig. S1) in *SMARCAL1* downregulated cells.

We will focus on c-*myc* transcription in this paper and explain how SMARCAL1 possibly regulates BRG1 in the companion paper.

### BRG1 and SMARCAL1 are present on the c-*myc* promoter

**Figure 1:**
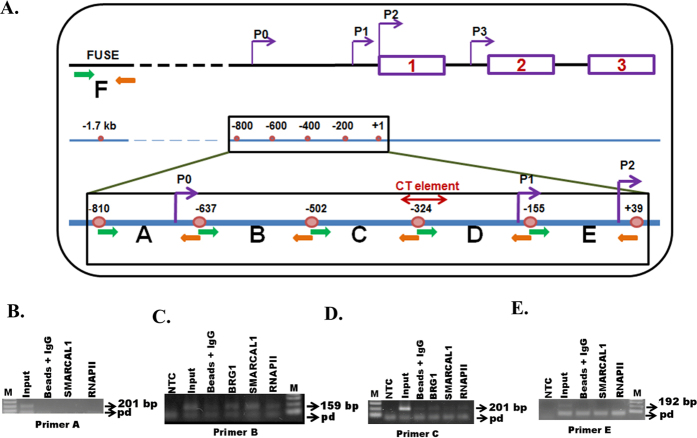
Analysis of occupancy of BRG1, SMARCAL1, and RNAPII on c-*myc* promoter. (**A**). Schematic representation of c-*myc* promoter showing promoters, P0, P1, P2, and P3 along with the CT element and FUSE region. The ChIP primers designed for analyzing the occupancy of BRG1, SMARCAL1 and RNAPII are also indicated in the figure. (**B**). Occupancy of SMARCAL1 and RNAPII at primer A region. (**C**). at primer B region, (**D**). at primer C region, and (**E**). at primer E region. The PCR products were resolved on 1% agarose gel. The primer-dimer formation is indicated by pd.

The above result indicated that either BRG1 or SMARCAL1 or both were possibly regulating c-*myc* transcription. Therefore, the occupancy of BRG1, SMARCAL1, and RNAPII on the c-*myc* promoter was probed using 5 pairs of overlapping primers (25-30 bp overlaps) designed with respect to the c-*myc* P2 promoter spanning the region from −810 bp to +39 bp each giving ~200 bp amplicon (Fig. 1A–E). We found that all three proteins were localized on the promoter at primer B and C region though the occupancy of SMARCAL1 appeared to be greater around the primer B region than the primer C region (Fig. 1C,D). The occupancy of BRG1 and RNAPII appeared to be similar around primer B and C regions (Fig. 1C,D).

Henceforth, we will refer to primer B and C regions as Myc_B_159_ and Myc_C_201_ respectively.

### Increased SMARCAL1 occupancy on c-*myc* promoter upon serum starvation

**Figure 2:**
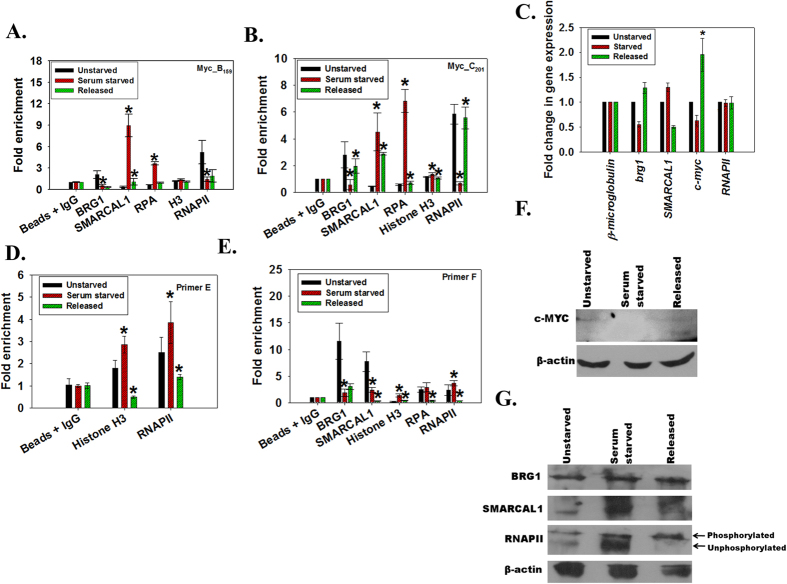
Analysis of occupancy of BRG1, SMARCAL1 and RNAPII on c-*myc* promoter during and after serum starvation. (**A**). Occupancy of BRG1, SMARCAL1, RPA, histone H3, and RNAPII was analyzed on Myc_B_159_ region using quantitative real-time RT-PCR following ChIP using respective antibodies. The star indicates statistical significance at p < 0.05. (**B**). Occupancy of BRG1, SMARCAL1, RPA, histone H3, and RNAPII on Myc_C_201_ region of c-*myc* promoter was analyzed using quantitative real-time RT-PCR following ChIP using respective antibodies. The star indicates statistical significance at p < 0.05. (**C**). Expression of c-*myc*, *SMARCAL1, brg1*, and *RNAPII* was monitored using quantitative real-time RT-PCR. *β- microglobulin* was used as control in this experiment. The star indicates statistical significance at p < 0.05. (**D**). Occupancy of histone H3 and RPA on Primer E region was analyzed using quantitative real-time RT-PCR following ChIP with respective antibodies. The star indicates statistical significance at p < 0.05. (**E**). Occupancy of BRG1, SMARCAL1, histone H3, RPA and RNAPII on FUSE region was analyzed using quantitative real-time RT-PCR following ChIP with respective antibodies. The star indicates statistical significance at p < 0.05. (**F**). c-Myc expression in unstarved, serum starved, and cells released from the block was analyzed using western blot. (**G**). SMARCAL1, BRG1, and RNAPII expression in unstarved, serum starved, and cells released from serum starvation were analyzed using western blots. In this experiment, β-actin was used as the control. Quantitation of pixel values is provided in Supplementary Fig. S2. Uncropped western blots are provided in Supplementary Fig. S11.

As both BRG1 and SMARCAL1 were present on the c-*myc* promoter, we wanted to know whether both the proteins were required simultaneously for c-*myc* transcription. It has been previously shown that c-*myc* transcription is reduced when cells are serum starved as they enter into G0 phase^29^. Upon release from serum starvation, c*-myc* transcription restarts as the protein is required for cells to enter into the cell cycle^29^. This experimental model of change in c-*myc* transcription upon serum starvation for 48 hours and subsequent release was, therefore, used to analyze the occupancy of SMARCAL1, BRG1, RNAPII, histone H3 and RPA on the c-*myc* promoter using chromatin immunoprecipitation. The analysis showed that occupancy of SMARCAL1 and RPA increased on the c-*myc* promoter increased both at Myc_B_159_ and Myc_C_201_ regions (Fig. 2A,B). Further, histone H3 occupancy increased 2-fold upon serum starvation around Myc_C_201_ as compared to the unstarved cells (Fig. 2B). Concomitantly, BRG1 and RNAPII occupancy on Myc_B_159_ and Myc_C_201_ regions decreased coinciding with decreased c-*myc* expression upon serum starvation (Fig. 2A–C). As RNAPII is known to stall at the proximal promoter region, we also examined the occupancy of RNAPII and H3 on the promoter P2 start site (Primer E in Fig.1). As expected, under serum starvation condition, RNAPII and H3 occupancy around promoter start site increased indicating that the polymerase was paused (Fig. 2D). Finally, we examined the occupancy of these proteins at the FUSE region as BRG1 is known to mediate its effect through this DNA segment. We found that occupancy of BRG1 and SMARCAL1 decreased on the FUSE region on serum starvation as compared to the unstarved cells (Fig. 2E). Further, at this region, the occupancy of histone H3 and RNAPII increased while that of RPA remained unchanged (Fig. 2E).

On release from serum starvation, SMARCAL1 occupancy decreased substantially on Myc_B_159_ as compared to Myc_C_201_ while the BRG1 occupancy started increasing on the Myc_C_201_ as compared to Myc_B_159_ (Fig. 2A,B). Further, RPA occupancy decreased on both Myc_B_159_ and Myc_C_201_ regions while H3 occupancy decreased on Myc_C_201_ region (Fig. 2A,B). Simultaneously, RNAPII occupancy on Myc_C_201_ increased 5-fold as compared to the serum starved condition, correlating with increased c*-myc* transcription (Fig. 2B,C). In addition, at the promoter start site, RNAPII and H3 occupancy decreased, indicating that the polymerase was no longer paused (Fig. 2D). The occupancy of SMARCAL1, RPA, H3, and RNAPII also decreased at the FUSE region while that of BRG1 increased slightly (Fig. 2E).

The transcript analysis showed that upon serum starvation the expression of *SMARCAL1* increased while that of c*-myc* decreased (Fig. 2C). We corroborated the transcript analysis with western blots. The expression of c-Myc decreased on serum starvation as compared to unstarved cells (Fig. 2F and Supplementary Fig. S2). However, the expression was not restored to unstarved cells on release from the block even though the transcript levels were upregulated (Fig. 2F and Supplementary Fig. S2). The expression of SMARCAL1 increased on serum starvation and decreased on release from the block while the expression of BRG1 was unchanged during and after serum starvation (Fig. 2G and Supplementary Fig. S2). The antibody for RNAPII recognizes both unphosphorylated and phosphorylated forms of the protein and we observed two bands in unstarved condition (Fig. 2G and Supplementary Fig. S2). The intensity of the lower band (unphosphorylated form) increased on serum starvation and decreased on release from serum starvation (Fig. 2G and Supplementary Fig. S2).

From this experiment we concluded that BRG1 was a positive regulator and SMARCAL1 was a negative regulator of c*-myc* transcription.

### BRG1 and SMARCAL1 direct their effects through c-*myc* promoter

To understand how BRG1 and SMARCAL1 were mediating their transcriptional regulation, the c*-myc* promoter (P2 TSS to −765 bp upstream) was cloned into pGL3 basic reporter plasmid. The construct was transfected into HeLa cells and the serum starvation experiment was performed. We found that on serum starvation the luciferase activity was downregulated by 15% and on release from serum starvation the luciferase activity was upregulated (Supplementary Fig. S2 and S3). To delineate the roles of Myc_B_159_ and Myc_C_201_ in transcription regulation, we cloned these DNA regions separately into pGL3 promoter plasmid and performed the same experiment (Supplementary Fig. S2). We found that the luciferase activity was downregulated when Myc_B_159_-promoter construct transfected HeLa cells were serum starved (Supplementary Fig. S2 and S3). This effect was not observed when Myc_C_201_ promoter was transfected into HeLa cells and serum starved, suggesting that SMARCAL1 primarily mediates its effect through Myc_B_159_ (Supplementary Fig. S2 and S3). Upon release from serum starvation, the luciferase activity remained downregulated in case of Myc_B_159_-promoter construct but was upregulated in case of Myc_C_201_-promoter construct, suggesting that BRG1 mediates its effect primarily through Myc_C_201_ region (Supplementary Fig. S2 and S3).

### The c-*myc* promoter accesibility is altered upon serum starvation

To understand the changes in the chromatin architecture of the c-*myc* promoter, the promoter region from −653 to −299 bp, with respect to P2 transcription start site, was scanned for protein occupancy in unstarved, serum starved, and released cells. We hypothesized that the DNA bound by protein would be inaccessible to micrococcal nuclease (MNase) in a manner similar to that of nucleosome-bound DNA whereas the protein-free region would be accessible for digestion by MNase. The protein-bound DNA after MNase digestion could, therefore, be purified and amplified using primers specific to the c-*myc* promoter (Supplementary Fig. S4). Comparing this result with the ChIP data would enable us to identify regions bound by BRG1, RNAPII, SMARCAL1, RPA and histone H3 in unstarved, serum starved, and released from starvation conditions on the c-*myc* promoter. In these experiments DNA of approximately 150 bp was purified, and therefore, proteins binding to a region larger or smaller than 150 bp would not be detected in this experiment (Supplementary Fig. S4). Finally, the protein occupancy was compared with respect to genomic DNA assuming that the genomic DNA was completely free of proteins as explained in methods.

**Figure 3:**
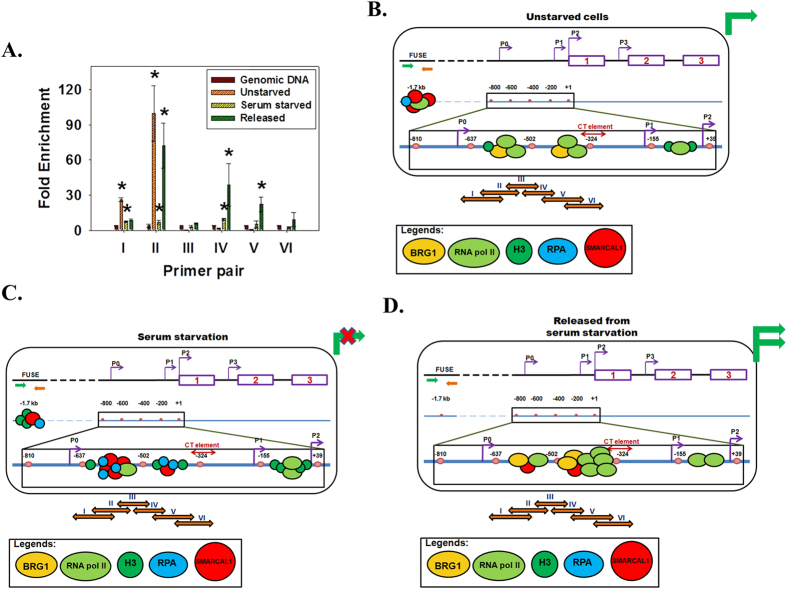
Protein occupancy on c-*myc* promoter is altered upon serum starvation. (**A**). Quantitative real-time RT-PCR was used to measure the fold enrichment of proteins on the c-*myc* promoter in unstarved HeLa cells, serum starved HeLa cells, and HeLa cells released from the serum starvation block. (**B**). Model explaining the occupancy of BRG1, SMARCAL1, RPA, H3, and RNAPII in unstarved HeLa cells; (**C**). in starved HeLa cells; (**D**). in cells released for 2 hours from 48 hours of serum starvation. The star indicates significant difference at p < 0.05.

We found that the protein occupancy on the c*-myc* promoter changed from unstarved to serum starved conditions and again in released condition. In unstarved condition, the regions mapped by primers I and II contained proteins while those mapped by primers III, IV, V, and VI were free of proteins (Fig. 3A). When cells were serum starved, the protein occupancy in the regions mapped by primers I and II decreased while it increased in regions scanned by primers IV and V (Fig. 3A) . When cells were released from serum starvation, protein occupancy on primer I region was unchanged with respect to starved conditions but increased in the region amplified by primer II, IV, V, and VI (Fig. 3A). Combining this data with the ChIP data enabled us to model BRG1, RNAPII, and SMARCAL1 on the promoter. In normal, unstarved conditions, BRG1 and RNAPII are present spanning −653 to −506 bp upstream of P2 (Fig. 3B). In addition, RNAPII and H3 are present at the transcription start site. Upon serum starvation, the protein occupancy changes and SMARCAL1, RPA and H3 are found present between −653 to −299 bp (Fig. 3C). Further, RNAPII and H3 occupancy around the transcription start site increases. When cells are released from serum starvation, BRG1 and RNAPII occupies −653 to −299 while SMARCAL1 is present from −523 to −299 bp (Fig. 3D). RPA and H3 are removed from Myc_B_159_ and Myc_C_201_ regions while RNAPII is no longer paused around the promoter proximal site (Fig. 3D).

### The c*-myc* promoter occupancy is altered in *SMARCAL1* downregulated cells

As stated earlier, c*-myc* expression was repressed in *SMARCAL1* downregulated cells. The same result was obtained when we transfected c*-myc* promoter cloned into pGL3 basic vector and measured the luciferase activity (Supplementary Fig. S5). Further, we measured the occupancy of RNAPII and H3K9Ac, a histone modification associated with transcription activation^30^, by ChIP and found that RNAPII occupancy on Myc_B_159_ and Myc_C_201_ was reduced in *SMARCAL1* downregulated cells (Supplementary Fig. S5). We also found that H3K9Ac levels were almost negligible in this region and there was no appreciable difference between the control and *SMARCAL1* downregulated cells (Supplementary Fig. S5). In addition, we hypothesized that downregulation of *brg1* should also result in reduced c*-myc* expression. Therefore, we also measured the c-*myc* transcript levels in Sh*brg1* cells and found them downregulated as expected (Supplementary Fig. S5).

Finally, we measured the promoter accessibility in control and *SMARCAL1* downregulated cells. As in the case of the unstarved cells, in control cells also the protein occupancy was found only in the regions amplified by primer pairs I and II (compare Fig. 3A and Supplementary Fig. S6). However, in case of *SMARCAL1* downregulated cells, the protein occupancy decreased in the regions amplified by these primer pairs (Supplementary Fig. S6). Further, there was no alteration in protein occupancy in the regions amplified by primer pairs III, IV, V, and VI, indicating that in the downregulated cells, the entire region spanning −653 to −299 bp was relatively free of protein occupancy (Supplementary Fig. S6).

Comparing this data with the ChIP enabled us to propose that RNAPII is present on the c-*myc* promoter in control cells while in *SMARCAL1* downregulated cells, this region is unprotected possibly because activating proteins are not present on the promoter resulting in decreased transcription (Supplementary Fig. S6).

c*-myc* expression is downregulated both in *SMARCAL1* downregulated cells as well as in serum starved cells. However, our model proposes that the mode of downregulation is different in these two cases. The expression of c*-myc* gene is downregulated in *SMARCAL1* downregulated cells because RNAPII is not present on the promoter. Under serum starvation condition, the c*-myc* expression is repressed because SMARCAL1 is present on the promoter region. Thus, these data led us to conclude that BRG1 and SMARCAL1 regulate c-*myc* expression in antagonistic manner.

### Myc_B_159_ DNA acts as an effector of ADAAD, the bovine homolog of SMARCAL1

As stated earlier, Baradaran-Heravi *et al*. have postulated that SMARCAL1 could possibly induce conformational changes in promoter regions^4^.

The QGRS^31^ software (http://bioinformatics.ramapo.edu/QGRS/analyze.php) predicted a G-quadruplex in Myc_B_159_region G2-L3-G2-L1-G2-L2-G2 where G is guanine and L is a loop comprised of any nucleotide (Supplementary Table S1). Human SMARCAL1 is difficult to overexpress and purify in sufficient amount for biophysical studies. Therefore, to understand whether SMARCAL1 can induce the formation of G-quadruplex in the Myc_B_159_ DNA, we used Active DNA-dependent ATPase A Domain (ADAAD) which is the bovine homolog of human SMARCAL1 (79% identity) and has been well-characterized in our laboratory^8^,^32^.

**Figure 4:**
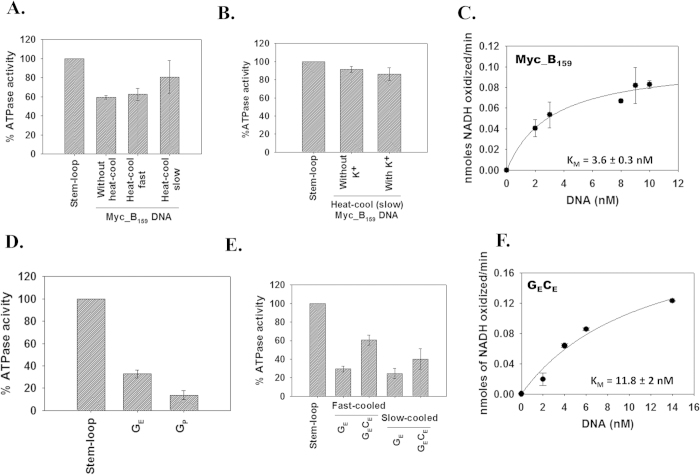
Myc_B_159_ as well as G_E_ are effectors for ADAAD. (**A**). ADAAD can hydrolyze ATP in the presence of Myc_B_159_. (**B**). Heat-cooled Myc_B_159_ DNA in the absence and presence of 100 mM K^+^ showed similar ATPase activity. In these experiments, 0.1 μM ADAAD and 20 nM DNA was used and the reaction was incubated at 37 °C for 90 minutes. The ATPase activity due to Myc_B_159_ DNA was compared with that due to stem-loop DNA. (**C**). The apparent K_M_ for ADAAD-Myc_B_159_ DNA was calculated using ATPase activity in presence of increasing concentration of DNA. The reaction was incubated at 37 °C for 45 minutes. (**D**). ADAAD prefers G_E_ as an effector compared to G_P_. In this experiment 2.4 μM ADAAD was incubated with 10 nM DNA for 45 minutes at 37 °C and the amount of ATP hydrolyzed was compared to that due to stem-loop DNA. (**E**). Comparison of effector properties of G_E_ and G_E_C_E_ with respect to stem-loop DNA. G_E_ and G_E_C_E_ was heat denatured and either rapidly cooled or slow-cooled before incubating with 0.24 μM ADAAD for 45 minutes at 37 °C and the amount of ATP hydrolyzed was compared to that due to stem-loop DNA. 10 nM DNA was used in the experiment. (**F**). The apparent K_M_ for ADAAD-G_E_C_E_ DNA was calculated using ATPase activity in presence of increasing concentration of DNA. The reaction was incubated at 37 °C for 45 minutes.

ADAAD was able to hydrolyze ATP in the presence of Myc_B_159_ (Fig. 4A). There was no significant difference in the amount of ATP hydrolyzed when Myc_B_159_ was heat-cooled either in the absence or presence of 100 mM K^+^ (Fig. 4A,B). The apparent K_M_ for ADAAD- Myc_B_159_ interaction was calculated to be 3.6 ± 0.3 nM (Fig. 4C).

### The interaction with the predicted G-quadruplex region is weaker than with Myc_B_159_

Next we sought to determine whether ADAAD specifically binds to the putative G-quadruplex forming region present within Myc_B_159_ DNA. For these studies, we synthesized G_E_, a 34 nt single-stranded DNA encompassing the predicted G-quadruplex sequence (Supplementary Table S2). G_E_ can form double-strand in presence of its complementary sequenc C_E_ (Supplementary Table S2). In addition, we also synthesized a single-stranded olignucleotide, G_P_, corresponding to the CT element, as a positive control since QGRS predicted that this region can also form a G-quadruplex. ATPase assays showed that G_E_ is a better effector than G_P_ (Fig. 4D).

Previously, we have shown that ADAAD specifically recognizes double-stranded to single-stranded transition regions in DNA molecule^8^. As both G_E_ and G_P_ have been predicted to form G-quadruplex, we used Mfold^33^ program to understand the differences in the secondary structure of G_E_ and G_P_. The Mfold program predicted that G_E_ can form a stem-loop structure with a 11 bp stem and 3 base loop (ΔG = −8.64 kcal/mol) while G_P_, the single-stranded oligonucleotide corresponding to the CT element, forms an unstable hairpin loop structure with the G_8_-T_18_ closing the loop (ΔG = 3.66 kcal/mol), thus providing a structural basis for the effector preference (Supplementary Fig. S7).

As under *in vivo* condition, DNA is present as a double-stranded molecule, we next compared the effector properties of single-stranded G_E_ molecule with that of the double-stranded G_E_C_E_ molecule. Further, the formation of stem-loop structure is an intramolecular event that can be brought about by denaturing the DNA and rapidly cooling it. In contrast, intermolecular events are favoured by cooling the DNA molecules slowly after denaturing. To understand whether the stem-loop structure is really critical for the interaction, we heated the DNA molecules and then either rapidly cooled it or slowly cooled it (Supplementary Fig. S8). We found that G_E_C_E_ was a better effector than G_E_ both when it was rapidly cooled and when it was slow cooled after heat denatuaration (Fig. 4E). Further, G_E_C_E_ was a better effector when it was fast cooled suggesting that the secondary structure of the oligonucleotide was essential for the interaction.

To understand whether ADAAD specifically recognizes G_E_C_E_ region within Myc_B_159_ DNA, we calculated the K_M_ for ADAAD-G_E_C_E_ interaction. As shown in Fig. 4F, the K_M_ was found to be 11.8 ± 2 nM, about 3-fold less than that for ADAAD-Myc_B_159_. However, the catalytic efficiency was higher for G_E_C_E_ as compared to Myc_B_159_ (Table 1). From this we concluded that though G_E_C_E_ can act as an effector of ADAAD, this is not sufficient for the interaction within the context of Myc_B_159_.

### Myc_B_159_ and G_E_C_E_ do not possess G-quadruplex structure

Circular dichroism has been used to monitor the conformational changes in DNA^34^. It has been reported that the CD spectra of anti-parallel G-quadruplex shows a negative peak at 260 nm and a positive peak at 290 nm while parallel G-quadruplex shows a positive peak at 260 nm and a negative peak at 240 nm. To understand whether double-stranded Myc_B_159_ and G_E_C_E_ can form G-quadruplex type structure in the absence of DNA and ATP, we used CD spectroscopy to analyze their structure. G_E_C_E_ after heat-cooling showed a negative peak at 230 nm while Myc_B_159_ showed a negative peak at 210 nm and a small rise at 275 nm, which are characteristic of double-stranded B-DNA (Supplementary Fig. S9).

As the G-quadruplex formation is an intramolecular event, we also analyzed the structure of single-stranded G_E_ DNA after heat denaturation and rapid cooling. The CD spectra showed a negative peak at 220 nm (Supplementary Fig. S9). This was similar to the peak obtained in case of stem-loop DNA (Supplementary Fig. S9).

The secondary structure, especially G-quadruplex formation, is dependent on monovalent cations, especially K^+^. Therefore, we analyzed the structures of G_E_C_E_, Myc_B_159_, G_E_, and stem-loop DNA in the presence of K^+^ and found the structure in the absence and presence of K^+^ were similar (Supplementary Fig. S9). Further, the structure of G_E_C_E_, G_E_ and stem-loop DNA in the presence of K^+^ was similar (Supplementary Fig. S9).

Finally, we analyzed the structure of G_P_ and found that it can indeed form the characteristic peaks of G-quadruplex (Supplementary Fig. S9).

From the CD analysis, we concluded that none of the DNA molecules at the concentration used in the experiment show the characteristic peaks of G-quadruplex either in the absence or presence of K^+^^34^.

### ADAAD induces a conformational changes on binding to the c*-myc* promoter

**Figure 5:**
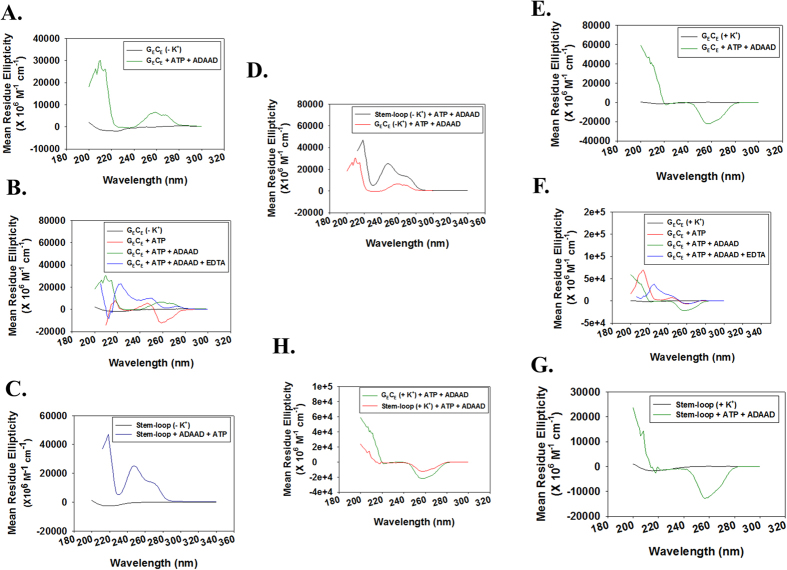
The conformation of G_E_C_E_ is altered in an ATP-dependent manner in the presence of ADAAD. (**A**). CD spectra of G_E_C_E_ (heat-cooled in the absence of K^+^) in the presence of ATP and ADAAD. (**B**). Comparison of CD spectra of G_E_C_E_ (heat-cooled in the absence of K^+^) in the presence of ATP alone, in the presence of both ATP and ADAAD, and after addition of EDTA. (**C**). CD spectra of stem-loop DNA (heat-cooled in the absence of K^+^) in the presence of both ATP and ADAAD. (**D**). Comparison of CD spectra of G_E_C_E_ and stem-loop DNA in the presence of both ATP and ADAAD. Both the DNA molecules were heat-cooled in the absence of K^+^. (**E**). CD spectra of G_E_C_E_ (heat-cooled in the presence of 100 mM K^+^) in the presence of ATP and ADAAD. (**F**). Comparison of CD spectra of G_E_C_E_ (heat-cooled in the presence of 100 mM K^+^) in the presence of ATP alone, in the presence of both ATP and ADAAD, and after addition of EDTA. (**G**). CD spectra of stem-loop DNA (heat-cooled in the presence of 100 mM K^+^) in the presence of both ATP and ADAAD. (**H**). Comparison of CD spectra of G_E_C_E_ and stem-loop DNA in the presence of both ATP and ADAAD. Both DNA molecules were heat-cooled in the presence of 100 mM K^+^. In these experiments, 0.5 μM DNA, 1 μM ADAAD, 2 mM ATP, 10 mM Mg^+2^, and 50 mM EDTA was used. The DNA molecules were heat denatured at 94 °C for 3 minutes and rapidly cooled to 4 °C either in the absence or presence of 100 mM K^+^.

Next, we analyzed the conformational changes induced in the double-stranded G_E_C_E_ and Myc_B_159_ DNA upon addition of ADAAD and ATP both in the absence and presence of K^+^ ions. When G_E_C_E_ (in the absence of K^+^) was incubated with both ADAAD and ATP, the DNA showed two positive peaks- one at 258 nm with a shoulder at 269 nm and a larger peak at 210 nm (Fig. 5A). Addition of EDTA to the reaction mix resulted in disruption of the conformational change, suggesting that ADAAD mediated ATP hydrolysis is necessary for the change in conformation of G_E_C_E_ (Fig. 5B). To understand whether the conformational was similar to the one induced in stem-loop DNA by ADAAD, we also analyzed the structure of stem-loop DNA in the absence of K^+^. As shown in Fig. 5C, the stem-loop DNA also showed two positive peaks–one at 250 nm and a shoulder at 272 nm and a larger peak at 215 nm (Fig. 5C,D).

Subsequently, we studied the effect of K^+^ ions on the conformational change induced in G_E_C_E_ DNA by the addition of ADAAD and ATP. In the presence of K^+^, we found that the G_E_C_E_ DNA showed a negative peak at 257 nm (Fig. 5E). The formation of this peak was also dependent on ATP hydrolysis as it was disrupted upon addtion of EDTA (Fig. 5F). Further, the stem-loop DNA also showed a negative peak at 257 nm in the when heat-cooled in the presence of K^+^ (Fig. 5G,H).

**Figure 6:**
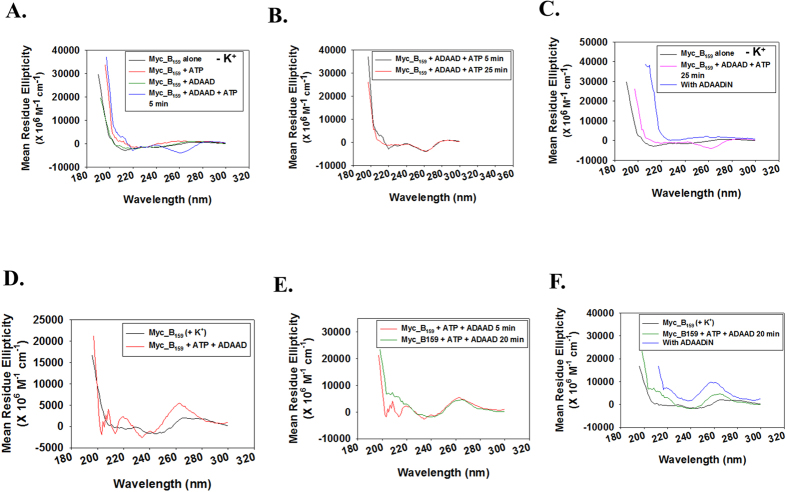
Conformational changes induced in Myc_B_159_ DNA. (**A**). Conformation of Myc_B_159_ DNA (heat-cooled in the absence of K^+^) in the presence of ATP and ADAAD. (**B**). Comparison of CD spectra of Myc_B_159_ DNA (heat-cooled in the absence of K^+^) in the presence of ATP and ADAAD at 5 minutes and 25 minutes. (**C**). CD spectra of Myc_B_159_ DNA (heat-cooled in the absence of K^+^) in the presence of ATP, ADAAD, and ADAADiN. (**D**). Conformation of Myc_B_159_ DNA (heat-cooled in the presence of 100 mM K^+^) in the presence of ATP and ADAAD. (**E**). Comparison of CD spectra of Myc_B_159_ DNA (heat-cooled in the presence of 100 mM K^+^) in the presence of ATP and ADAAD at 5 minutes and 20 minutes. (**E**). CD spectra of Myc_B_159_ DNA (heat-cooled in the presence of 100 mM K^+^) in the presence of ATP, ADAAD, and ADAADiN. In these experiments 0.15 μM Myc_B_159_ DNA, 0.1 μM ADAAD, 0.5 mM ATP, 10 mM Mg^+2^ and 5 μM ADAADiN were used.

Finally, we studied the conformational change induced in Myc_B_159_ when incubated with ADAAD and ATP. This DNA, however, in the absence of K^+^ showed a negative peak at 262 nm, which did not change with incubation at 37 °C (Fig. 6A,B). Formation of this peak was dependent on the presence of both ADAAD and ATP (Fig. 6A). The conformation was disrupted when ADAADiN, the known inhibitor of ADAAD^35^,^36^ was added to the reaction, indicating continued hydrolysis is needed for maintaining the conformation (Fig. 6C). In contrast, when Myc_B_159_ heat-cooled in the presence of K^+^ was incubated with ADAAD and ATP, a positive peak at 262 nm was obtained which did not change with incubation at 37 °C and was dependent on the presence of both ADAAD and ATP (Fig. 6D,E) . Addition of ADAADiN, however, resulted in stabilization of the positive 262 nm peak (Fig. 6F).

From these experiments we conclude that ADAAD, the bovine homolog of SMARCAL1, can induce conformational change in the c*-myc* promoter DNA in an ATP-dependent manner such that structure so formed acts as an impediment to RNAPII binding, thus leading to transcription repression.

### Inverse correlation between expression of SMARCAL1 and c-MYC

What is the physiological relevance of SMARCAL1 regulating c-*myc* expression? An analysis of c-*myc*^37^ and *SMARCAL1*^5^ levels in adult mouse tissues showed that the expression of *SMARCAL1* was high in kidney, brain, and liver while c*-myc* was not expressed in these tissues though it was expressed in the newborn mice. As symptoms of SIOD include renal dysfunction and cereberal ischemia, we hypothesized that SMARCAL1 expression during differentiation is critical for repressing c*-myc* expression.

Differentiation of K562 into myeloid cell lineage in response to PMA treatment has been well-documented and correlates with transcription repression of c-*myc*^38^,^39^,^40^. We used this system to understand whether downregulation of c-*myc* expression during differentiation was dependent on SMARCAL1 expression.

K562 cells were treated with PMA for upto 5 days and the expression of *brg1*, *SMARCAL1*, and *c-myc* were estimated using quantitative real-time RT-PCR. As shown in Supplementary Fig. S10, *c-myc* expression was downregulated and *SMARCAL1* expression was upregulated after treatment with PMA validating the inverse correlation we observed between *SMARCAL1* and c*-myc* expression when cells were serum starved and subsequently, released from the block (Fig. 2C). Interestingly, *brg1* expression was unaltered when K562 cells differentiated.

## Discussion

The transcription factor, c-*myc*, regulates 10–15% genes in the mammalian cells^18^. The expression of c-*myc* itself is regulated by exquisite machinery consisting of DNA secondary structures, transcription factors, ATP-dependent chromatin remodeling factors, and multiple promoters^23^.

The CT element present 150 bp upstream of promoter P1 has been shown to form G-quadruplex and i-motif structures^24^,^25^,^26^,^41^. Nucleolin has been shown to bind to this element both *in vitro* and *in vivo*^42^. Further, the protein has been shown to induce G-quadruplex structure in this element *in vitro* suggesting that it might be doing the same *in vivo*^42^. In addition to the CT element, the promoter also contains a FUSE region about 1.7 kb upstream of the promoter P2^23^. This region possesses nucleosomes and BRG1 has been shown to reposition the nucleosomes during activation of c-*myc* expression^29^.

In this paper, we have identified yet another element regulating the expression of c-*myc*. SMARCAL1, a distant member of the ATP-dependent chromatin remodeling protein family, has been shown to function as an annealing helicase required for stabilizing replication forks when DNA is damaged in the S phase^9^,^10^,^11^,^12^,^13^,^14^,^43^. Mutations in SMARCAL1 have been correlated with SIOD, a pleiotropic disorder characterized by spondyloepiphyseal dysplasia, renal dysfunction and T-cell immunodeficiency^15^,^44^. As SIOD encompasses many different organ systems, it has been hypothesized that SMARCAL1 might function as a transcriptional regulator of a particular subset of genes, possibly through its chromatin remodeling activity^15^. SIOD patients have been found to show disturbed gene expression profiles necessary for skeletal development, renal tissue maintenance and T-cell development^16^. However, till now SMARCAL1 has not been shown to localize at promoters and there exists only one report that documents the interaction of SMARCAL1 with histones^45^.

Using a combination of *in vivo* and *in vitro* experiments we have shown that SMARCAL1 regulates c*-myc* transcription. The protein binds to a region termed as Myc_B_159_ present upstream of the CT element. This region has not previously been shown to form either G-quadruplex or to regulate the transcription activity. Our studies show that under normal conditions BRG1 and RNAPII bind to this region allowing for transcription to occur. During serum starvation, when cells enter into the G0 phase and c-*myc* transcription is shut off, SMARCAL1 occupancy on Myc_B_159_ increases while that of BRG1 and RNAPII decreases correlating with transcription repression. When cells are released from the block, the occupancy of SMARCAL1 decreases while that of BRG1 and RNAPII increases resulting in transcription activation. Thus, BRG1 functions as a positive regulator while SMARCAL1 acts as a negative regulator of c*-myc* transcription.

SMARCAL1 has been shown to interact with RPA and we find that these two proteins are present on the c*-myc* promoter on serum starvation. However, from these experiments it is not clear whether RPA recruits SMARCAL1 to the promoter or not. Further, our experiments also show that the Myc_B_159_ and Myc_C_201_ regions are free from nucleosomes under normal conditions. When cells were serum starved, H3 occupancy increased on Myc_C_201_ as well as at the transcription start site (primer E region) indicating SMARCAL1 might induce formation of an inaccessible chromatin structure leading to transcription repression.

Using the bovine homolog of SMARCAL1 and *in vitro* assays we have found that Myc_B_159_ is indeed an effector of ATPase activity and the protein binds to this region with an apparent K_M_ of 3.6 ± 0.3 nM, which was similar to the stem-loop DNA that we reported earlier as the best effector for this protein^8^. Bioinformatic^31^ studies predicted a potential G-quadruplex forming region within Myc_B_159_. A 34-nucleotide double-stranded DNA, called G_E_C_E_, spanning the potential G-quadruplex was also found to be an effector of ADAAD, though the K_M_ values indicated that the interaction is 3-fold weaker, suggesting the protein recognizes additional regions within Myc_B_159_ DNA.

CD spectroscopy showed that interaction of ADAAD with G_E_C_E_ in the presence of ATP leads to a conformational change in the DNA, which was similar to the one induced in stem-loop DNA leading us to hypothesize SMARCAL1 might be recognizing c*-myc* promoter region due to its structural features viz. ability to form stem-loop structure. The conformation of Myc_B_159_ adopted in the presence of ADAAD and ATP is different from that of G_E_C_E_. This difference between Myc_B_159_ and G_E_C_E_ could be due to the length of the molecule. Despite the difference in the spectra we can conclude that binding of ADAAD results in a conformation change in DNA which possibly impedes the binding of RNAPII and BRG1 to the promoter resulting in transcription repression.

Using the K562 cell lines as a model system, we have shown that during differentiation the expression of *SMARCAL1 i*s upregulated while that of c*-myc* is downregulated, suggesting a negative correlation between the expression of these two proteins.

However, we have also shown in the paper that downregulation of *SMARCAL1* leads to downregulation of c*-myc*. This is due to the fact that downregulation of *SMARCAL1* leads to downregulation of *brg1* also as a positive feedback loop governs the levels of these two proteins (Haokip *et al*. Transcriptional regulation of ATP-dependent chromatin remodeling factors: positive feedback loop regulates the levels of SMARCAL1 and BRG1). As BRG1 is a positive regulator of c-*myc* expression, we observe a downregulation in the levels of c*-myc* in *SMARCAL1* downregulated cells. The positive feedback regulation between SMARCAL1 and BRG1, however, seems to be operative only under specific conditions. For example, when K562 cells undergo differentiation, the expression of BRG1 does not change while SMARCAL1 is significantly upregulated. Similarly, during serum starvation, SMARCAL1 expression is upregulated but not that of BRG1. The upregulation of SMARCAL1 expression correlates with downregulation of c-*myc* expression during both K562 differentiation and serum starvation of HeLa cells.

The relevance of regulation of c*-myc* expression by SMARCAL1 became clear when we compared northern blot analysis of these c*-myc* and *SMARCAL1* in mouse adult tissues reported by Zimmerman *et al*., and Coleman *et al*., respectively^5^,^37^. c-*myc* expression was absent in tissues where *SMARCAL1* was expressed at high level. Interestingly, in kidney and brain tissues, c*-myc* was not expressed in adult mice^37^, while *SMARCAL1* was expressed^5^. Further, lung and spleen tissues show low *SMARCAL1* expression that correlates with high c-*myc* expression. Renal dysfunctions as well as impaired brain development are symptoms observed in SIOD patients^15^,^46^. We, therefore, hypothesize that some of the symptoms observed in SIOD patients might be due to the inability of the mutant SMARCAL1 to regulate c*-myc* expression.

This is the first report of SMARCAL1 regulating c*-myc* expression. Many questions are unanswered. For example, what is the conformational change effected in the promoter region and how does it block RNAPII interaction. Further, how is SMARCAL1 recruited to the promoter? In the companion paper, we show that SMARCAL1 can interact with both RNAPII and histone H3. It is, thus, possible that the protein is recruited via interaction with a specific modification present on histones or it is recruited via RPA as it happens during DNA repair. We also do not know the protein partners that interact with SMARCAL1 for regulating transcription. We also do not know how many genes SMARCAL1 regulates. Further experiments would enable us to answer these questions.

## Methods

### Reagents

Dulbecco’s Modified Eagle’s Medium (DMEM), penicillin-streptomycin cocktail, amphotericin B, sodium bicarbonate, TRIzol reagent, Hoechst 33342, and Escort transfection reagent were purchased from Sigma-Aldrich (USA). Fetal bovine serum was purchased from Gibco (USA). Restriction enzymes, Turbofect, M-MuLV Reverse Transcriptase kit, Hi-fidelity PCR enzyme mix, FastAP thermo sensitive Alkaline Phosphatase and the INSTAclone TA-cloning kit were purchased either from MBI Fermentas (USA) or from NEB (USA). QIAquick gel extraction kit was purchased from Qiagen (USA). Protein A-CL agarose bead resin was purchased from Bangalore Genei (India). 2X SYBR Green PCR master mix, micro-amp Fast 96-well reaction plates (0.1 ml) and micro-amp optical adhesive films were purchased either from Applied Biosystems (USA) or Kapa Biosystems (USA). For western blotting, Immobilon-P PVDF membrane was purchased from Merck-Millipore (USA). X-ray films, developer, and fixer were from Kodak (USA).

### Antibodies

The various primary antibodies, unless otherwise mentioned, were purchased from Sigma-Aldrich (USA), Cell Signalling Technology (USA), Abcam (UK) or Bangalore Genei (India). The HRP-conjugated anti-mouse IgG and anti-rabbit IgG antibodies were obtained from Bangalore Genei (India). The catalog # of antibodies used in this study are as follows: RNAPII (Rpb1 CTD, Cell Signaling Technology, Cat #2629; 1: 6500 dilution), c-MYC (Santa Cruz, sc-40; 1: 2500), H3 (Cell Signaling Technology, Cat #3638), RPA (Cell Signaling Technology, Cat #2208), BRG1 (Sigma Aldrich, Cat #B8184: 1: 5000), and β-actin (Sigma Aldrich, Cat #A1978: 1: 10,000 dilution). SMARCAL1 antibody (1: 1800 dilution) was raised against the N-terminus HARP domain as discussed previously^35^.

### Primers

The list of primers used in cloning, quantitative real-time RT-PCR, ChIP and protein occupancy assay is given in Supplementary Table S3-6 respectively. All oligonucleotides were synthesized by Sigma-Aldrich (USA).

### Cell culture and transfection

HeLa and K562 cells obtained from NCCS were cultured in Dulbecco’s modified Eagle’s medium and RPMI1640 respectively supplemented with 10% fetal bovine serum and 1% antibiotic cocktail. HeLa cells seeded to a confluency of 50–70% were transfected with various plasmid constructs.

### Preparation of *SMARCAL1* downregulated cell line

HeLa cells were seeded at 50–70% confluency and transfected with ShRNA clones obtained from Sigma Aldrich (USA). Stably transfected cells were selected using DMEM supplemented with 2 μg/ml puromycin. These cells, termed as Sh, were further subjected to clonal selection and three clones-Sh1, Sh2, and Sh3 were obtained.

### Construction of pGL3-*myc* promoter construct

The c-*myc* promoter was amplified from HeLa genomic DNA using specific primers with Hi-fidelity PCR enzyme. The 765 bp amplified product was cloned into pTZ57R/T vector. The clone was confirmed by restriction digestion followed by sequencing. c-*myc* promoter region was then released from the T/A clone and cloned into pGL3 basic vector using SacI and NheI restriction sites. The construct was confirmed using restriction digestion and used for further analysis.

### Cloning of Myc_B_159_ and Myc_C_201_ in pGL3 promoter vector

The Myc_B_159_ and Myc_C_201_ regions were amplified with Pfu DNA polymerase using pGL3-*myc* promoter as template with specific primers. The amplification products were digested with SacI/KpnI and were cloned into similarly double-digested pGL3 promoter vector. The constructs were confirmed using restriction digestion and used for further analysis.

### RNA isolation and cDNA preparation

Total RNA was extracted using the TRIzol reagent (Sigma-Aldrich). 90% confluent cells in a 35 mm plate were lysed with 1 ml of the TRIzol reagent to give a homogenized lysate. The lysate was transferred to a tube. 200 μl of chloroform was added to each tube per ml of TRIzol reagent, shaken vigorously and allowed to stand for 10–15 minutes at room temperature. The samples were centrifuged at 11,000 rpm for 15 minutes at 4 °C. The top aqueous layer obtained was transferred to a fresh tube and 0.5 ml of isopropanol was added per ml of TRIzol reagent, mixed and allowed to stand at room temperature for 10–15 minutes. The samples were then centrifuged at 11,000 rpm for 10 minutes at 4 °C. The RNA pellet obtained was washed with 70% ethanol and resuspended in DEPC-treated water. RNA concentrations were determined using NanoDrop 2000 (Thermo Fisher Scientific, USA) and equal amount of RNA from various samples was used for preparing cDNA using random hexamer primers according to the manufacturer’s protocol. The prepared cDNA was checked for quality by performing a PCR using suitable primers.

### Quantitative real-time RT-PCR

Quantitative real-time RT-PCR was performed with 7500 Fast Real-Time PCR system (ABI Biosystems, USA) using gene-specific primers designed for exon-exon junctions. For each reaction15 μl of samples were prepared in triplicates and the data obtained was analyzed using Fast7500 software provided by manufacturer. The p-value was calculated using Sigma-Plot (Sigma-Plot, USA).

### Cell extract preparation for western blot

Cell extracts were made using either RIPA lysis buffer (50 mM Tris-Cl pH 7.5, 300 mM NaCl, 2 mM EDTA, 1% v/v NP-40, 0.5% w/v sodium deoxycholate, 1% w/v sodium dodecyl sulphate) or urea lysis buffer (90% 8.8 M urea, 2% 5 M NaH_2_PO_4_ and 8% 1 M Tris-Cl pH 8.0). Briefly, cells were grown in 100 mm culture dishes to a confluency of 75–80%, harvested and thoroughly washed thrice with PBS. The cells were pelleted at 2500 rpm for 10 minutes at 4 °C and then resuspended in the appropriate lysis buffer. The cells were incubated on ice for 15 minutes with regular mild tapping followed by sonication (5 cycles of 30 sec on/off). The sonicated cell suspension was spun at 13000 rpm for 10 minutes at 4 °C. The supernatant was collected and used for further experiments. The protein concentration was determined using Bradford reagent.

### Chromatin Immunoprecipitation (ChIP)

ChIP was performed according to the X-ChIP protocol provided online by Abcam (http://www.abcam.com/ps/pdf/protocols/x_CHip_protocol.pdf) with minor modifications. Briefly, cells were cross-linked by adding formaldehyde (final concentration 1%) and later quenched by adding glycine (final concentration 125 mM) to the media. The cells were then washed thoroughly using ice-cold PBS, scraped into 1 ml PBS and collected in eppendorf tubes. Cells were pelleted at 2500 rpm at 4°C for 10 minutes. The pelleted cells were treated with freshly prepared lysis buffer (10 mM Tris-Cl, pH 8.0; 140 mM NaCl; 1 mM EDTA, pH 8.0; 1% Triton-X100; 0.1% sodium deoxycholate; 0.1% sodium dodecyl sulphate, 1 mM PMSF and protease inhibitor cocktail 10 μl/plate) for 10 minutes at 4°C followed by sonication using a water-sonicater (40 cycles of 30 sec pulse/ 20 sec rest). The sonicated samples were centrifuged and the supernatant was used for further analysis. 50 μl of the sonicated sample was purified and the DNA concentration was determined. A small part of the purified DNA was run on 1.5% agarose gel to check for sonication efficiency. The remaining DNA was stored to be used as the “Input” sample. An equal amount of chromatin was taken for performing IP using various antibodies. One sample was kept as Beads-IgG negative control. Pre-adsorbed protein A bead resin (pre-adsorbed with 75 ng/μl sonicated salmon sperm DNA and 0.1 μg/μl of BSA) and 5 μg of the desired antibody was added to each sample. The cocktails were incubated overnight at 4 °C on an eppendorf-rotator. This was followed by washing the pelleted bead resin 3 times in wash buffer (0.1% (w/v) sodium dodecyl sulphate; 1% Triton X-100; 2 mM EDTA, pH 8.0; 150 mM NaCl; 20 mM Tris-Cl, pH 8.0) and once in final wash buffer (0.1% sodium dodecyl sulphate; 1% Triton X-100; 2 mM EDTA, pH 8.0; 500 mM NaCl; 20 mM Tris-Cl, pH 8.0). The bound DNA was eluted using fresh elution buffer (1% sodium dodecyl sulphate; 100 mM NaHCO_3_). The eluted DNA was purified using phenol-chloroform and precipitated as mentioned in protocol. The resuspended DNA was used for ChIP PCR using standardized primers.

### Serum starvation assay

HeLa cells were cultured in DMEM with 10% FBS. For starvation assay, cells were cultures with 0.4% serum for 48 hours. Cells were released from serum starvation by adding 10% FBS and harvested at indicated time points for analysis.

### Differentiation of K562 using PMA

K562 cells were cultured in RPMI1640 media containing 10% FBS and 1% antibiotic cocktail. For PMA-induced differentiation, cells were treated with 10 nM PMA for atleast 24 hours. As a control, cells were mock-treated with DMSO.

### Promoter accessibility assay

The assay was executed using the method described by Infante *et al*.^47^. Briefly, HeLa cells grown to 2 × 10^7^ to 3 × 10^7^ cells were cross-linked with formaldehyde (1% (v/v) final concentration) for 15 min at room temperature and later quenched with glycine (125 mM final concentration). Cells were then washed with 10 ml cold PBS twice at 4 °C, scraped out, resuspended in NPS buffer (0.5 mM spermidine, 0.075% NP-40, 50 mM NaCl, 10 mM Tris-Cl, pH 7.5, 5 mM MgCl_2_, 1 mM CaCl_2_, and 1 mM β-mercaptoethanol) and digested with 30 units of micrococcal nuclease for 15 min at 37 °C. Digestions were stopped by shifting the tubes to 4 °C and adding EDTA and EGTA to final concentrations of 15 mM and 2.3 mM respectively. Subsequently, the digested samples were treated with 60 μl 10% (v/v) SDS, 10 mg/ml proteinase K and 10 μl of 10 mg/ml RNase for 15 min at 37 °C. The DNA was extracted twice with phenol saturated with 0.1 M Tris-Cl, pH 7.5 and once with equal volume of chloroform. The DNA was precipitated with 0.1 volume of 3 M NaOAc, pH 5.3 and 2.5 volume of 100% ice-cold ethanol. The precipitated DNA was resuspended in TE pH 8.0 buffer and analyzed on a 1.5% agarose gel. The mono nucleosomes were purified from the gel and subjected to RT- PCR using primers designed for the c-*myc* promoter region (Supplementary Table S6).

The amount of protein-protected DNA for each primer pair was measured as a ratio between MNase digested and undigested genomic DNA and then normalization was done with the highest amount of protein protected DNA. We have assumed that the genomic DNA purified from the cells was devoid of proteins and therefore, this DNA was not digested with MNase. Each experiment was done in triplicates and average data along with standard deviation has been reported. Further, the p-value (Sigma-Plot, USA) was calculated to determine the statistical significance.

### Dual-luciferase reporter assay

Equal numbers of HeLa cells were seeded in 12-well plates and co-transfected, with pGL3-c*-myc* promoter or pGL3-empty vector and pRL-TK, using turbofect. The luciferase assay was performed 36 hours after transfection using the Dual-luciferase reporter assay kit (Promega) and the luciferase activity was measured and normalized with respect to the controls.

### ATPase assays

ATPase assays were performed using purified ADAAD as published previously^32^.

### CD spectra

CD spectra were recorded using Chirascan (Applied Photophysics). Briefly, CD spectra of DNA were recorded in 1 mM sodium phosphate buffer (pH 7.0) in the presence of ATP, Mg^+2^, and ADAAD. The concentrations of these reagents are indicated in the figure legends. For each experiment, 5 scans were taken at 37 °C. Spectra of appropriate buffer conditions were also taken at each time point. The spectra reading for each condition was subtracted from the appropriate buffer reading and plotted as a function of the wavelength. The CD values were converted to Mean Residue Ellipticity [θ] using the following equation:


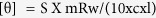


Where S is the CD signal, c is the concentration of DNA in M, l is pathlength in cm, and mRw is the mean residue weight given by mRw = molecular weight of the oligonucleotide/number of bases in the oligonucleotide.

For ATPase assays as well as for CD spectra, Myc_B_159_ was amplified using appropriate primers. The amplicon was agarose gel-purified and used in these experiments. DNA fragments used for the assays were either used directly without any heat/cool treatment or were heated at 95 °C for 3 minutes followed by slow/fast cooling. For assays requiring K^+^, 1 M KCl was added to the purified DNA to a final concentration of 100 mM.

## Additional Information

**How to cite this article**: Sharma, T. *et al*. SMARCAL1 negatively regulates c-myc transcription by altering the conformation of the promoter region. *Sci. Rep*. **5**, 17910; doi: 10.1038/srep17910 (2015).

## Supplementary Material

Supplementary Information

## Figures and Tables

**Table 1:** Comparison of kinetic parameters for ADAAD interaction with Myc_B_159_ and G_E_C_E_ DNA.

DNA	V_max_ (nmoles of NADH oxidized/min)	K_M_ (nM)	k_cat_ (s^-1^)	k_cat_/K_M_(X 10^7^ M^-1^ s^-1^)
**Myc_B**_**159**_	0.11 ± 0.02	3.6 ± 0.3	0.075 ± 0.02	2.12 ± 0.6
**G**_**E**_**C**_**E**_	10.45 ± 0.9	11.75 ± 1.9	2.89 ± 0.24	24.7 ± 2.0
