# Supplementary material for: SMARCAL1 Negatively Regulates C-Myc Transcription By Altering The Conformation Of The Promoter Region
Source: Sci Rep. 2015 Dec 9;5:17910. doi: 10.1038/srep17910 (PMC4673416; doi:10.1038/srep17910)
Supplement: Supplementary Information [file srep17910-s1.doc]

**SUPPLEMENTARY INFORMATION**

**SMARCAL1 NEGATIVELY REGULATES C-*MYC* TRANSCRIPTION BY ALTERING THE CONFORMATION OF THE PROMOTER REGION**

Tapan Sharma, Ritu Bansal, Dominic Thangminlen Haokip, Isha Goel and Rohini Muthuswami**^*^**

School of Life Sciences, JNU, New Delhi 110067

**^*^To whom correspondence should be addressed:** Rohini Muthuswami, Room # 333, School of Life Sciences, JNU, New Delhi 110067. Phone number + 91 11 26704154; Email: [rohini_m@mail.jnu.ac.in](mailto:rohini_m@mail.jnu.ac.in)

**
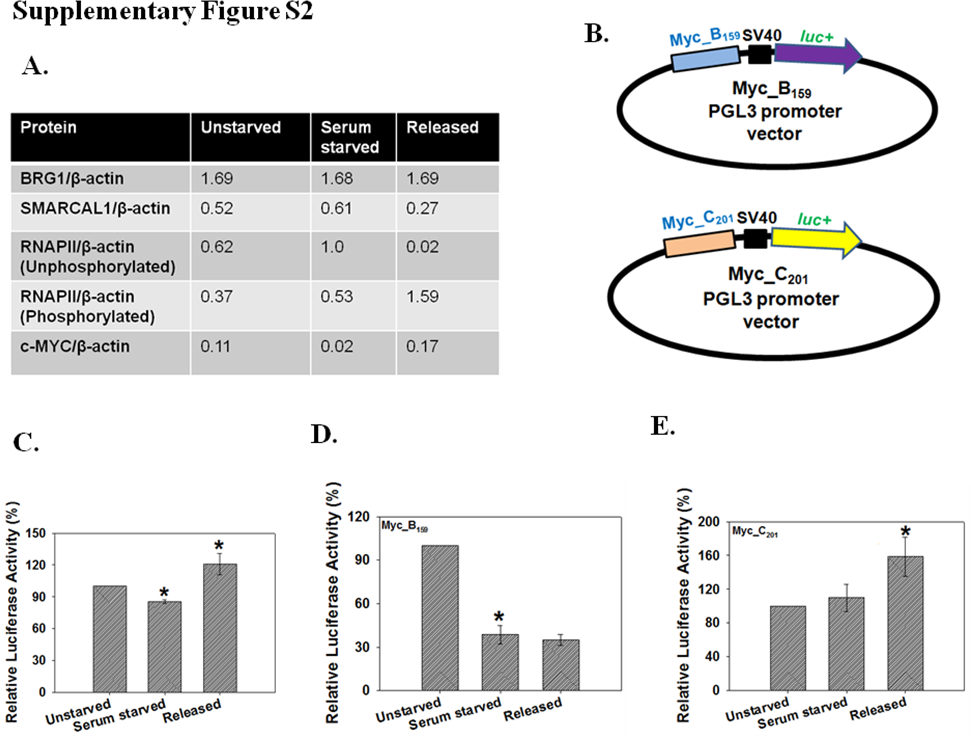
**


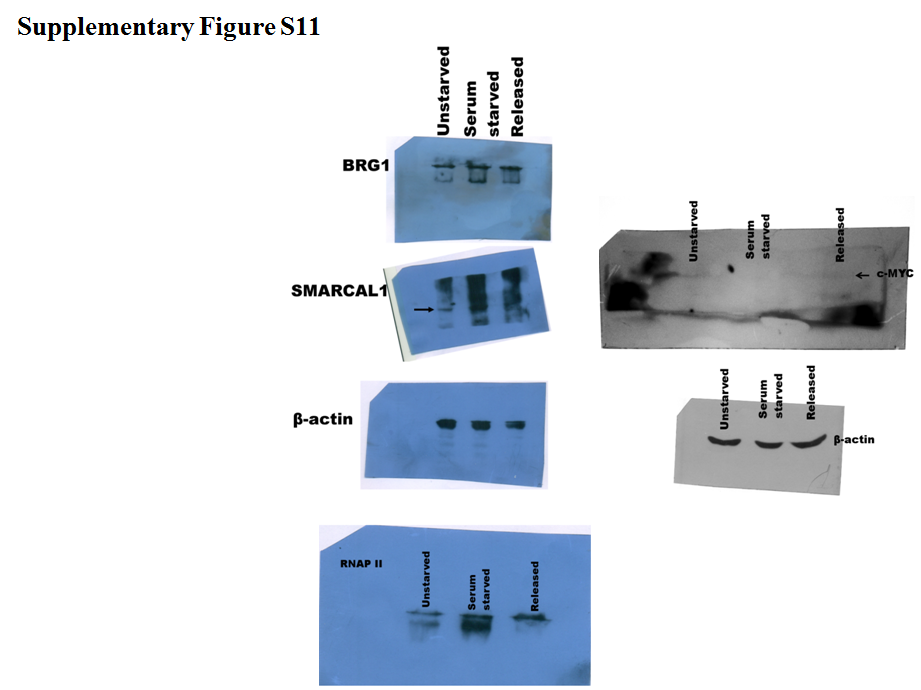


**Supplementary Table S1. Sequence of Myc_B_159_ region on c*-myc* promoter where SMARCAL1 occupancy was found.**

| Forward strand 5′🡪3′ | Reverse strand 5′🡪3′ |
| --- | --- |
| CCTCCACTCTCCCTGGGACTCTTGATCAAAGCGCGGCCCTTTCCCCAGCCTTAGCGAGGCGCCCTGCAGCCTGGTACGCGCGT***GG*CGT*GG*C*GG*TG*GG***CGCGCAGTGCGTTCTCGGTGTGGAGGGCAGCTGTTCCGCCTGCGATGATTTATACTCACAGG | CCTGTGAGTATAAATCATCGCAGGCGGAACAGCTGCCCTCCACACCGAGAACGCACTGCGCGCCCACCGCCACGCCACGCGCGTACCAGGCTGCAGGGCGCCTCGCTAAGGCTGGGGAAAGGGCCGCGCTTTGATCAAGAGTCCCAGGGAGAGTGGAGG |

The sequence highlighted in bold and underlined was shown by QGRS mapper to form G-quadruplex.

**Supplementary Table S2. Sequence of G_E_, G_P_, C_E_, and stem-loop oligonucleotides used in our study. The putative G-quadruplex forming sequence is in bold.**

| **Oligonucleotide** | **Sequence (**5′🡪3′) | **Nucleotide length (nt)** | **Formation of G-quadruplex**^1^ | **Formation of stem-loop structure**^2^ |
| --- | --- | --- | --- | --- |
| G_E_ | CGCGCGT***GG*CGT*GG*C*GG*TG*GG***CGCGCAGTGCGTT | 34 | Yes; score 19 | Yes; ΔG = -8.64 kcal/mol |
| G_p_ | ***GGG*GAGGGTG*GGG*AGGGTG*GGG*AAGGTG*GGG***AGG | 34 | Yes; score 42 | Yes; ΔG = 3.55 kcal/mol |
| Stem-loop | GCGCAATTGCGCTCGACGATTTTTTAGCGCAATTGCGC | 38 | No | Yes; ΔG = -16.36 kcal/mol |
| C_E_ | AACGCACTGCGCGCCCACCGCCACGCCACGCGCG | 34 | No | Yes; ΔG = -5.74 kcal/mol |

G_E_ and C_E_ are complementary to each other and can form double-stranded DNA.

**References:**

1. Kikin, O., D’Antonio, L. & Bagga, P. S. QGRS Mapper: a web-based server for predicting G-quadruplexes in nucleotide sequences. *Nucleic Acids Res.* **34,** W676–W682 (2006).

2. Zuker, M. Mfold web server for nucleic acid folding and hybridization prediction. *Nucleic Acids Res.* **31,** 3406–3415 (2003).

**Supplementary Table S3. List of primers used in cloning.**

| **Gene** | **Forward primer (5′à3′)** | **Reverse primer (5′à3′)** |
| --- | --- | --- |
| c-*myc* promoter | CGAGAGCTCCCCAACAAATGCAATGGGAGTT | CGAGCTAGCAGCCTCTGAGGAGCCCTGCCCT |
| *myc_B_159_* | ATAGGTACCCTCCACTCTCCCTGGGACTCTT | ATAGAGCTCCCTGTGAGTATAAATCATCGCA |
| *myc_C_201_* | CTGCGATGATTTATACTCACAGGAC | AGGTGGGGAGGAGACTCAGCCGGG |

**Supplementary Table S4.** List of primers used for RT-PCR.

| **Gene** | **Forward primer (5′🡪3′)** | **Reverse primer (5′🡪3′)** |
| --- | --- | --- |
| *gapdh* | CGGAGTCAACGGATTTGGTCGTAT | GGAACATGTAAACCATGTAGTTGAGG |
| *SMARCAL1* | CACCAAGGACAAAACTAAACAGCAGCAG | GTCCAAGATATATTCAATGACAGATGGGA |
| c-*myc* | CCGACGCGGGGAGGCTATTC | CGCGGGAGGCTGCTGGTTTT |
| *β-microglobulin* | GAGGCTATCCAGCGTACTCCAAAG | GTGTAGTACAAGAGATAGAAAGACC |
| *RPB1* | TTGTGGACTCTAACAACCCAAAG | CACCATGGCCCTTTTCTTTGGTC |

**Supplementary Table S5. List of primers used for ChIP experiments**.

| **Amplicon size (bp)** | **Annealing temperature (^o^C)** | **Forward primer (5'→3')** | **Reverse primer (5'→3')** |
| --- | --- | --- | --- |
| Primer A region (201) | 64 | CCCAACAAATGCAATGGGAGTTTATTCA | TCAAGAGTCCCAGGGAGAGTGGAGG |
| Primer B region (159) | 59 | CCTCCACTCTCCCTGGGACTCTTGA | CCTGTGAGTATAAATCATCGCAGG |
| Primer C region (201) | 58.5 | CTGCGATGATTTATACTCACAGGAC | AGGTGGGGAGGAGACTCAGCCGGG |
| Primer E region (192) | 70.5 | GCTGTGCTGCTCGCGGCCGCCACCG | CGCTCCCTCTGCCTCTCGCTGGAAT |
| Primer F  FUSE region (232) | 63 | TAAAGCTGAATTGTGCAGTGCATCG | AACATTCTTCTCATCCTTGGTCCCT |

**Supplementary Table S6. List of primers used in promoter accessibility assay.**

| **Amplicon size (bp)** | **Annealing temperature (^o^C)** | **Forward primer (5'→3')** | **Reverse primer (5'→3')** |
| --- | --- | --- | --- |
| Primer I  (128) | 55 | CTTCTTTCCTCCACTCTCCCTGGGA | TCCACACCGAGAACGCACTG |
| Primer II  (99) | 58 | CAGCCTTAGCGAGGCGCCCTGC | CATCGCAGGCGGAACAGCTG |
| Primer III  (104) | 55 | CAGTGCGTTCTCGGTGTGGA | GTGCTGCTCCTCCGTAGCAGT |
| Primer IV  (120) | 58 | CTGTTCCGCCTGCGATGATTTATAC | CTACCATTTTCTTTTGCTCCCTCTG |
| Primer V  (102) | 58 | GGGAGCAAAAGAAAATGGTAGGCGCGC | GGAGACTCAGCCGGGCAGCCGA |
| Primer VI  (102) | 55 | CTCTCTTACTCTGTTTACATCCTAG | AGGGGCGCTTATGGGGAGGGT |

**SUPPLEMENTARY FIGURE LEGENDS**

**Supplementary Figure S1. Analysis of SMARCAL1, BRG1 and c-*myc* expression in *SMARCAL1* downregulated cells.** (A). Sh*SMARCAL1* cells were subjected to clonal selection and three monoclonals Sh1, Sh2 and Sh3 were obtained. Transcript levels of *SMARCAL1* in *SMARCAL1* downregulated cells. The star indicates statistical significance at p<0.001. (B). Transcript levels of *SMARCAL1* in polyclonal Sh *SMARCAL1* cells. The star indicates statistical significance at p<0.001. (C). Transcript levels of *brg1* in *SMARCAL1* downregulated cells. The star indicates statistical significance at p<0.001. (D). Transcript levels of *brg1* in polyclonal Sh *SMARCAL1* cells. The star indicates statistical significance at p<0.001. (E). Transcript levels of c-*myc* in *SMARCAL1* downregulated cells. The star indicates statistical significance at p<0.001. (F). Transcript levels of *c-myc* in polyclonal Sh *SMARCAL1* cells. The star indicates statistical significance at p<0.001.

**Supplementary Figure S2.** **BRG1 and SMARCAL1 direct their effects through c-*myc* promoter.** (A). Quantitation of pixel values of western blots shown in Figure 2F and 2G. (B). Model showing the cloning of Myc_B_159_ and Myc_C_201_ into pGL3 promoter vector. These construct enabled us to assess the potential of these two regions to modulate transcription as measured by luciferase activity. (C). pGL3-c-myc promoter construct was transfected into HeLa cells and the expression of luciferase was measured during and after release from serum starvation. The star indicates statistical significance at p<0.05. (D). pGL3-Myc_B_159_ promoter construct was transfected into HeLa cells and luciferase expression was monitored during and after release from serum starvation. The star indicates statistical significance at p<0.05. (E). pGL3-Myc_C_201_ promoter construct was transfected into HeLa cells and luciferase expression was monitored during and after release from serum starvation. The star indicates statistical significance at p<0.05.

**Supplementary Figure S3. Model explaining the role of Myc_B_159_ and Myc_C_201_ in modulating transcription of c*-myc* gene.** (A). During unstarved condition, BRG1 and RNAPII bind to Myc_B_159_ and switch on transcription. When cells are serum starved, SMARCAL1 binds to Myc_B_159_ and shuts down transcription. On release from serum starvation, SMARCAL1, BRG1, and RNAPII are bound to Myc_B_159_. However, transcription does not restart. (B). Under normal conditions, BRG1 and RNAPII bind to Myc_C_201_ to switch on transcription. During serum starvation, SMARCAL1 is bound to Myc_C_201_ but transcription is not switched off. When cells are released from serum starvation, SMARCAL1, BRG1, and RNAPII are bound to Myc_C_201_ and transcription is upregulated. From this it is clear that Myc_B_159_ acts as a repressive element while Myc_C_201_ is a positive regulator of transcription.

**Supplementary Figure S4. Promoter Accessibility Assay.** (A).Schematic representation of the primers used in promoter accessibility on the c-*myc* promoter. (B). Representative agarose gel showing the digested chromatin. Lanes M is DNA marker; Lane 1 shows digestion of chromatin using 10 U of MNase; Lane 2 shows chromatin digested with 20 U of MNase; Lane 3 shows chromatin digested with 30 U of MNase. All digestions were done at 37^o^C for 30 minutes. For all further experiments described, chromatin was digested for 30 minutes at 37^o^C using 30 U of MNase.

**Supplementary Figure S5. BRG1 positively regulates c-*myc* expression.** (A). Luciferase activity was measured in control and *SMARCAL1*downregulated (Sh) cells after transfecting them with pGL3-c-*myc* promoter construct. The star indicates statistical significance at p<0.05. (B). RNAPII and H3K9Ac occupancy at primer B and C regions of c-*myc* promoter in control and *SMARCAL1*downregulated (Sh) cells was analyzed by resolving the PCR products on 1% agarose gel. (C). Quantitative real time-PCR was used to quantitate the occupancy of RNAPII and H3K9Ac on Myc_B_159_ region of c-*myc* promoter. The star indicates statistical significance at p<0.05. (D). Quantitative real time-PCR was used to quantitate the occupancy of RNAPII and H3K9Ac on Myc_C_201_ region of c-*myc* promoter. . The star indicates statistical significance at p<0.05. (E). c-*myc* expression was measured in *brg1* downregulated cells using quantitative real time RT-PCR.

**Supplementary Figure S6. Protein occupancy on c-*myc* promoter is altered in *SMARCAL1* downregulated cells.** (A). Quantitative real time-PCR was used to measure the fold enrichment of proteins on the c-*myc* promoter in control and SMARCAL1 downregulated (Sh) cells. The star indicates significant difference at p<0.05. (B). Model explaining the occupancy of RNAPII in control HeLa cells. (C). Occupancy of RNAPII on c-*myc* promoter in SMARCAL1 downregulated cells. The ChIP data as well as the occupancy assay showed that the c-*myc* promoter was devoid of proteins in SMARCAL1 downregulated cells.

**Supplementary Figure S7. Structure predication using Mfold software**. (A). G_E_ oligonucleotide. (B). G_P_ oligonucleotide. (C). Stem-loop DNA.

**Supplementary Figure S8. Schematic representation of formation of intra- and inter-molecular DNA secondary structures on heat-cooling.**

**Supplementary Figure S9. CD spectra of DNA in the absence of ADAAD and ATP.** (A). CD spectra of G_E_C_E_ DNA in the absence and presence of 100 mM K^+^. (B). CD spectra of Myc_B_159_ DNA in the absence and presence of 100 mM K^+^. (C). CD spectra of G_E_ DNA in the absence and presence of 100 mM K^+^. (D). CD spectra of stem-loop DNA in the absence and presence of 100 mM K^+^. (E). Comparison of CD spectra of G_E_C_E_, G_E_ and stem-loop DNA in the presence of 100 mM K^+^. (E). CD spectra of 5 µM single-stranded G_P_ DNA. G_P_ DNA was used as positive control as it has been reported to form G-quadruplexes.

0.5 µM of G_E_C_E_, G_E_, stem-loop and Gp oligonucleotides and 0.15 µM of Myc_B_159_ was used for recording the CD spectra.

**Supplementary Figure S10.** **Inverse correlation between *SMARCAL1* and *c-myc* expression on differentiation of K562 cells.** Expression of *brg1*, *SMARCAL1*, and c*-myc* was estimated using quantitative real time-PCR after treatment with PMA for indicated time periods. The differences were significant at P < 0.001.

**Supplementary Figure S11. Uncropped western blots** showing the expression of SMARCAL1, BRG1, RNAPII, and c- MYC in unstarved, serum starved, and released HeLa cells. β-actin is used as control in these experiments.
